# Lysozyme Modification Using Proteolytic Enzymes

**DOI:** 10.3390/molecules28176260

**Published:** 2023-08-26

**Authors:** Łukasz Tomczyk, Grzegorz Leśnierowski, Renata Cegielska-Radziejewska

**Affiliations:** Department of Food Quality and Safety Management, Poznan University of Life Sciences, 60-624 Poznan, Poland; grzegorz.lesnierowski@up.poznan.pl (G.L.); renata.cegielska-radziejewska@up.poznan.pl (R.C.-R.)

**Keywords:** lysozyme, bioactive peptides, enzymatic hydrolysis, hydrolytic activity, antimicrobial activity, hydrophobic activity, antioxidant activity

## Abstract

The lysozyme in the chicken egg white consists of various bioactive amino acids. However, these compounds are inactive when they are in the sequence of parent proteins. They become active only when isolated from these proteins. The aim of this study was to modify lysozyme with proteolytic enzymes under specific conditions of the reaction environment so as to obtain active biopeptides. The physicochemical properties of the resulting preparations were also assessed. Our study showed that the modification of lysozyme with hydrolytic enzymes (pepsin and trypsin) under strictly specified conditions resulted in obtaining biopeptide preparations with new and valuable properties, as compared with native lysozyme. After the enzymatic modification of lysozyme, two structural fractions were distinguished in the composition of the resulting preparations—the monomeric fraction and the peptide fraction. The modified lysozyme exhibited high surface hydrophobicity and high total antibacterial activity despite the decrease in the hydrolytic activity. Modification of lysozyme with hydrolytic enzymes, especially pepsin, resulted in preparations with very good antioxidative properties.

## 1. Introduction

Lysozyme obtained from the protein fraction of a chicken egg consists of 129 amino acids. This protein with antimicrobial properties has a molecular mass of 14.3 kDa [1,2]. Lysozyme hydrolyses the glycosidic bonds between N-acetylglucosamine and N-acetylmuramic acid in the polysaccharides, forming the cell walls of bacteria. It also hydrolyses the glycosidic bonds in chitin [3]. Due to the antibacterial properties of lysozyme, this enzyme has high application potential in the food, pharmaceutical, cosmetic, and veterinary industries. It is an adjuvant applied in the treatment of various bacterial infections, diseases of the skin, eyes, and mouth, as well as in cancer [4,5,6]. However, due to the specific structure of this native enzyme, its antibacterial effect is limited only to Gram-positive bacteria [1,7]. It is Gram-negative bacteria that constitute a significant proportion of both pathogenic microorganisms and the microbes causing food spoilage during storage. Lysozyme can also be effective against these bacteria, but it must be adapted to a form that will be active against them. Research has proved that this effect can be obtained by modifying the enzyme to a dimeric form and/or to a form in which the hydrophobic surface originally closed inside the enzyme molecule will be brought to its surface, and its active centre will be opened [8]. As a result of such modifications, new products are formed—these are bioactive lysozyme derivatives with completely new physicochemical and antibacterial properties and, thus, with a new application potential. Among the known types of lysozyme modifications, the best-known and most frequently used are those that cause oligomerisation and increase the hydrophobic surface [8,9]. Researchers increasingly often make attempts to break down the lysozyme monomer into smaller molecules. [10].

Bioactive peptides can be isolated using enzymatic hydrolysis combined with thermochemical methods [11]. Pepsin is one of the most common enzymes used for enzymatic hydrolysis of parent proteins. The pH of the enzymatic hydrolysis environment plays an important role in this process [12,13,14]. Apart from pepsin, another enzyme used for the enzymatic hydrolysis of lysozyme is trypsin, usually in hydrolytic systems together with pepsin and papain [15,16].

The aim of this study was to modify lysozyme with proteolytic enzymes under specific conditions of the reaction environment so as to obtain active biopeptides. The physicochemical properties of the resulting preparations were also assessed.

## 2. Results

The aim of this study was to obtain bioactive peptides from lysozyme isolated from chicken egg whites by applying thermo-enzymatic hydrolysis with pepsin and trypsin. The resulting preparations were analysed to assess the effect of the applied modification procedures on their quality, i.e., the fractional composition, hydrolytic activity, changes in the surface hydrophobicity and antioxidative activity.

**Figure 1:**
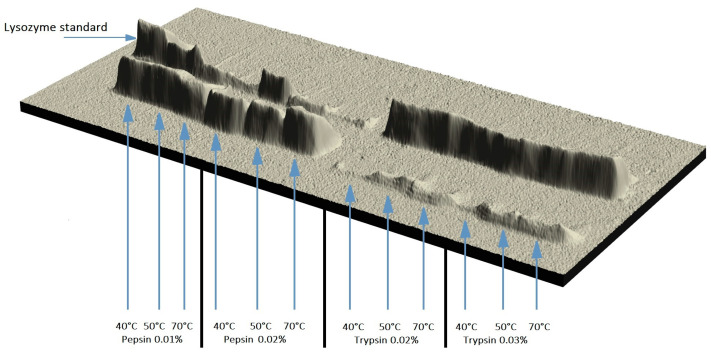
A 3D image of the electrophoretic separation of modified lysozyme preparations at 40, 50, and 70 °C with pepsin concentrations of 0.01% and 0.02% and trypsin concentrations of 0.02% and 0.03%.

In the first stage of the research (variant 1), the optimal conditions for modifications conducted in an acidic environment (pH = 2.0) were determined so as to effectively release lysozyme peptides. The test results are shown in Table 1. Figure 1 shows the results of electrophoretic analysis in the form of a 3D image, which illustrates the development of a new form of lysozyme depending on the conditions of its modification.

The enzymatic modification of lysozyme with proteolytic enzymes, i.e., pepsin and trypsin, always resulted in the monomeric fraction and peptide fraction, regardless of the modification conditions. The analysis of the results showed that pepsin released peptides more effectively. The temperature of the modification process significantly influenced the formation of peptides. The modification temperature of 70 °C resulted in the highest degree of hydrolysis of the lysozyme monomer in the samples, whereas the lowest degree was observed at 40 °C. The pepsin concentrations were of lesser importance. The statistical analysis did not reveal any significant differences between the modifications conducted with different pepsin concentrations at any temperature of the process. The number of biopetides in the samples modified with trypsin was always statistically lower than in those modified with pepsin. Similarly to pepsin, the amount of peptides formed in the modification conducted with trypsin depended on the process temperature rather than the concentration of this enzyme in the modified samples. The concentration of both enzymes, i.e., pepsin and trypsin, in the modified solution only influenced the amount of new lysozyme forms.

The analysis of the successive effects of the modifications showed that the type of the proteolytic enzyme and the modification temperature significantly affected the changes in the surface hydrophobicity of the modified lysozyme as well as its hydrolytic and antioxidative activity. The results of the statistical analysis showed that the efficiency of hydrolysis was determined using the temperature of the process at 70 °C preparations with the highest antioxidative activity and the greatest increase in surface hydrophobicity were obtained. At the same time, they were characterised by high hydrolytic activity. The hydrolysis with pepsin at 70 °C increased the surface hydrophobicity by 34–45%, whereas trypsin increased it by only 3–11%, as compared with the native enzyme. In contrast to pepsin, trypsin had a much weaker effect on the structural changes of the lysozyme molecule responsible for moving its hydrophobic interior to the surface of the enzyme. On the other hand, the use of trypsin resulted in higher antioxidative activity than the use of pepsin (Table 1). At the same time, the research showed that regardless of the enzyme used in the modification process, the resulting preparations always had stronger antioxidative properties than the native form of the enzyme. The modification processes were not indifferent to the hydrolytic activity of lysozyme. Trypsin had a weaker effect on the lysozyme molecule and resulted in the smallest changes. Regardless of the concentration of trypsin and the temperature of the modification process, despite the slight decrease in the hydrolytic activity, it remained high and was only slightly lower than the activity of the native enzyme (Table 1). On the other hand, similarly to hydrophobic changes, pepsin significantly reduced the hydrolytic activity. The higher the pepsin concentration and the higher the modification temperature, the lower the hydrolytic activity (Table 1). It is noteworthy that this phenomenon was always observed during various lysozyme modifications, especially thermal modifications. The antibacterial properties of the enzyme were usually not reduced. On the contrary, the total antibacterial activity of lysozyme was very often higher than that of the native enzyme, and it was also effective against Gram-negative bacteria [8].

The results of the first stage of the research on the enzymatic modification of lysozyme, as well as the data provided in reference publications, indicated that the acidity of the environment and the duration of hydrolysis significantly affected the enzyme modification [8,17,18]. The research was continued (variant 2) to determine the influence of these factors on the enzymatic modification of lysozyme. In this variant, native lysozyme was modified with pepsin at a concentration of 0.01% and temperature of 70 °C, i.e., the optimal conditions resulting from variant 1 of the study. The modification was conducted under the changing conditions of environment acidity (pH 2.0, 3.0, and 4.0) and process duration (1, 2, and 3 h). The lysozyme modification process was conducted according to the procedure described in Section 4.1.

**Figure 2:**
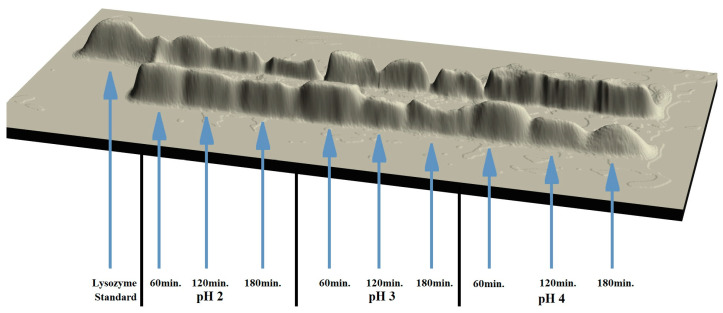
A 3D image of the electrophoretic separation of lysozyme preparations modified at 70 °C and at different pH variants, i.e., 2, 3 and 4 for 60, 120, and 180 min.

Like in the first variant of the study, as a result of the modification of lysozyme, its modified forms were obtained. These were preparations containing both a monomer and a peptide fraction. The preparations were analysed. The results of the analysis are shown in Table 2. The results of the electrophoretic analysis are shown in Figure 2.

The analysis of the results showed that the effects of lysozyme modification significantly depended on the conditions of the process. The longer duration of the modification process had a negative influence on its effectiveness (Table 2). As the duration of the modification process increased, the percentage content of peptides decreased regardless of the pH of the environment. One-hour modification of the enzyme in an acidic environment (pH = 2.0) resulted in the highest percentage of peptides, i.e., 67.9%. The lowest percentage of the peptide fraction, i.e., 21.1%, was obtained after 3 h modification in an environment with pH = 4.0. As the duration of the modification process increased, the average decrease in the content of the peptide fraction in the samples modified at pH = 2.0 and 3.0 amounted to 17.82% and 17.13%, respectively, whereas in the samples modified at pH= 4.0 it was 51.97%.

The study also showed that the acidity of the environment and the duration of the modification process significantly influenced changes in the hydrophobicity of the enzyme. The highest average increase in surface hydrophobicity, i.e., 34.0%, was observed in the sample modified for one hour in an environment with pH = 2.0, whereas the lowest increase, i.e., 10.6%, was found in the sample modified for three hours in an environment with pH = 4.0. As can be seen, the amount of released peptides and their content in a given preparation were of key significance for the increase in surface hydrophobicity. The higher the percentage of peptides there were, the higher the surface hydrophobicity was. The release of the peptides exposed the hydrophobic interior of the lysozyme to its surface.

The new modification conditions used in the second variant of the experiment also significantly influenced the values of the other physicochemical parameters, i.e., the hydrolytic and antioxidative activity of the modified lysozyme. The highest antioxidative activity, i.e., 113.4 TE/mgL on average, was observed in the preparations obtained after three-hour modification in an environment with pH = 4.0. The lowest antioxidative activity was measured in the preparations modified for one hour at pH = 4.0. The opposite dependence was observed for the hydrolytic activity. Its highest value was observed under the least drastic conditions of the modification process, i.e., one-hour duration and pH = 4.0 (Table 2).

It is necessary to make an extensive microbial analysis in order to fully assess the antibacterial activity of enzyme-modified lysozyme. The results of this analysis will be provided in a separate study. Preliminary tests were conducted in this study to assess the effect of the preparations obtained after the modification of lysosome with pepsin and trypsin on selected types of bacteria. The tests showed that the effect of the modified lysozyme on bacteria depended on the modification method. The samples obtained during the second modification variant (Variant 2) were characterised by better antibacterial activity. Table 3 shows the results of an evaluation of the antibacterial effect of selected pepsin-modified lysozyme preparations containing the highest proportion of the peptide fraction. The incubation of the modified lysozyme with the bacterial strains used in the tests prolonged the lag phase of the bacteria. The lysosome preparations had a better antibacterial effect against the Gram-positive *Listeria innocua* bacteria. Neither the trypsin-modified lysozyme nor the modified lysozyme preparations obtained in the first variant of the experiment (variant 1) inhibited bacterial growth effectively. However, the results of the preliminary test of the enzymatically modified lysozyme indicated that the spectrum of the antibacterial activity of the enzyme could be extended. An in-depth assessment will be possible only after conducting a full study on the antibacterial activity of the enzyme, which is currently being conducted independently.

## 3. Discussion

Lysozyme contained in the chicken egg white is the subject of numerous scientific studies because it has various beneficial properties. Native lysozyme occurs in the form of a monomer. If there are favourable environmental conditions, it becomes dimerised or even oligomerised [8]. The main aim of our study was to achieve the opposite effect, i.e., to obtain shorter amino acid chains in the form of released bioactive lysozyme peptides and to assess the physicochemical properties of the resulting preparations. Appropriate analytical techniques were applied to investigate the range of changes occurring in lysozyme as a result of its thermo-enzymatic modification conducted with pepsin and trypsin in various environmental conditions. Earlier studies showed that bioactive peptides were released as a result of the enzymatic modification of lysozyme with pepsin [14,19]. Our research showed that it is also possible to obtain such preparations by the modification of lysozyme with proteolytic enzymes in a strongly acidic environment. The best hydrolytic effect was observed after the addition of pepsin, whereas trypsin hydrolysed lysozyme to a lesser extent. This effect could be expected, as the data provided in some publications suggested that trypsin worked most effectively in combination with other enzymes, e.g., pepsin or papain. Such hydrolytic systems usually resulted in significant or even complete hydrolysis of lysozyme and the release of its bioactive peptides [15,16].

The analysis of changes in the hydrophobicity of enzymatically modified lysozyme clearly showed that the hydrolysis of the enzyme caused an increase in its surface hydrophobicity, as was the case with other lysozyme modifications [2,8,20]. This means that during such modifications, the internal hydrophobic part of the enzyme is also exposed to its surface [8]. According to Ibrahim’s theory [21,22], conformational changes of the enzyme take place and expose the hydrophobic sites of various amino acids, thereby increasing the hydrophobic surface of the lysozyme. Regardless of the mechanism, each increase in the surface hydrophobicity and a simultaneous decrease in the hydrolytic activity of the enzyme [8] always caused an increase in the total antibacterial activity of lysozyme. A similar effect was expected and observed in our study, as evidenced by the microbial tests. Apart from the destructive effect on Gram-positive bacteria, the preparations obtained after the lysozyme modification also exhibited an antibacterial effect against Gram-negative bacteria.

The preparations obtained as a result of the enzymatic modification of lysozyme with pepsin and trypsin were also characterised by a significantly higher antioxidative activity than that of the native enzyme. This effect was also observed by other researchers, who found that biopetides obtained from lysozyme were always characterised by increased antioxidative activity [13,15,23,24]. In our study, the lysozyme modification conditions for the highest antioxidative activity were developed. At the same time, the increase in the antioxidative activity was closely correlated with the applied temperature and the duration of the modification process.

## 4. Materials and Methods

A commercial lysozyme monomer preparation with an activity of 21,252 U/mg, isolated from chicken egg white (Belovo, Belgium), was used as the research material.

### 4.1. Lysozyme Modification

There were two variants of the enzymatic modification of lysozyme.

1. The first variant consisted of hydrolytic modification of lysozyme with pepsin and trypsin. Two types of 3% aqueous lysozyme solutions (pH = 2) containing 0.01% and 0.02% of pepsin and 0.02% and 0.03% of trypsin were prepared for modification. This solution was prepared for modification by adjusting its pH to 2.0 with hydrochloric acid (with the appropriate concentration: 1.0 or 0.5 M). A lysozyme solution without enzymes was used as a reference sample. The pepsin and trypsin samples, as well as the reference sample, were modified in a BUCHI Syncore^®^ analytical reactor (Switzerland) at 40, 55, and 70 °C for 60 min. Then, the reaction was stopped by heating the samples for 5 min at 85 °C and then, after cooling, their pH was set at 7.0 with sodium hydroxide.
2. The second variant consisted of an assessment of the effect of the acidity of the environment and the duration of the modification. For this purpose, subsequent modifications of lysozyme were conducted at the optimal concentration of both enzymes and the optimal modification temperature obtained in the first variant of the experiment. This time, the modifications lasted 60, 120, and 180 min, and they were conducted in an environment with pH of 2.0, 3.0, and 4.0.

The resulting preparations were frozen and then lyophilised in a Labconco freeze dryer (USA). Next, they were analysed.

### 4.2. Electrophoresis

The electrophoretic analysis was conducted in accordance with the method described by Leśnierowski (2007) [25]. The process was conducted in acrylamide gel containing 15% acrylamide separating gel and 6% stacking gel. Samples were dissolved in sample buffer (0.3% TRIS-HCl (pH 6.8), 30% glycerol, 0.1% bromophenol blue, 6% SDS) and heated at 100 °C for 5 min. 3 µL of the samples was applied onto the stacking gel. The electrophoretic separation was conducted at a current of 60 mA for the stacking gel and 90 mA for the separating gel. When the electrophoresis front approached the end of the separating gel, the separation was terminated, and the obtained gels were fixed for 30 min in a solution containing the appropriate proportions of methanol, acetic acid, and water (5:4:1). The gels were stained with 0.025% Coomassie brilliant blue R solution for 20 h. Then, the gels were decolorised in an aqueous solution of acetic acid (10%) until their background was completely discoloured. The finished gels were scanned. The electropherograms were stored as computer files and subjected to densitometric analysis in order to determine the quantitative share of individual fractions in the preparations obtained after the modification. The TotalLab Quant software 1.5.170 (Nonlinear Dynamics Ltd., Gosforth, UK) was used for the densitometry.

### 4.3. Hydrolytic Activity

The spectrophotometric method described by Leśniowski (2007) [25] was used to measure the hydrolytic activity of the obtained preparations. The method consists of determining the reduction in turbidity in a bacterial suspension (*Micrococcus lysodeikticus*) after adding lysozyme to it. The analysis was conducted under strictly defined measurement conditions, including the acidity of the environment, the measurement temperature, the volume of the sample, the amount of bacteria added, as well as the wavelength and the optical path length. The analysis was conducted with a VSU2-P Carl Zeiss Jena spectrophotometer (VSU2-P, Oberkochen, Germany).

### 4.4. Hydrophobicity of Modified Lysozyme

The hydrophobicity of lysozyme was measured spectrophotometrically, with the method developed by Lieske and Konrad (1994) [26] and modified by Leśnierowski (2007) [26]. 8-anilinonaphthalene-1-sulfonic acid (ANS, Sigma-Aldrich, Munich, Germany) and an LS 55 luminescence spectrometer (Perkin Elmer, Norwalk, CT, USA) with an output wavelength λ = 390 nm and an emitter wavelength λ = 470 nm were used for the measurements. Lysozyme solutions (0.01%) and adequate dilutions were prepared in phosphate buffer (pH 6.0). 3 mL of each dilution and 15 µL of ANS dissolved in methanol were collected. After 15 s, the fluorescence intensity was measured. The hydrophobicity of the surface was equal to the slope coefficient of the fluorescence intensity vs the protein concentration curve.

### 4.5. Antioxidative Activity

The antioxidative activity of the preparations was analysed with the ABTS method described by Re et al. 1999 [27]. The results were expressed as the ability of the antioxidants to scavenge ABTS radicals in relation to the ability of Trolox (Sigma-Aldrich, Munich, Germany). They were expressed as mM Trolox equivalent per millilitre of sample (mM TE/mL).

### 4.6. Microbiological Test

The antibacterial activity of the monomer and modified lysozyme preparations against selected Gram-positive (*Listeria innocua* DSM 20649) and Gram-negative bacterial strains (*Escherichia coli* PCM 2793, *Proteus mirabilis* PCM 1361, *Salmonella enteritidis* PCM 941) (Hirszfeld Institute of Immunology and Experimental Therapy Wrocław, Poland) was assessed. The bacteria were cultured for 24 h. Next, the bacterial suspensions were prepared in 0.85% NaCl (Biomérieux) with a density of 0.5 (the McFarland scale) in a Densimat apparatus (Biomérieux). Then, decimal dilutions were prepared: for *Listeria innocua* bacteria—107 CFU/mL, for other bacterial strains—105 CFU/mL. Simultaneously, lysozyme solutions with concentrations of 0.0625%, 0.125%, 0.25%, 0.5%, 1%, 1.5%, and 3% in sterile water were prepared. The optical density of lysozyme and bacteria was measured with Bioscreen C. The samples analysed in the experiment were made from 30 µL of the bacterial inoculum, 150 µL of lysozyme solution, and 120 µL of nutrient broth. Then, the samples were incubated at 37 °C for 72 h. Changes in the optical density of the sample during the experiment were recorded automatically every 30 min for 44 h.

### 4.7. Statistical Analysis

The results were analysed statistically with the Statistica 13.1 software and expressed as mean values. Analysis of variance (ANOVA) was applied to examine the differences between the variables.

## 5. Conclusions

Our study showed that the modification of lysozyme with hydrolytic enzymes, especially pepsin (but also trypsin), under strictly specified conditions resulted in obtaining biopeptide preparations with new and valuable properties, as compared with native lysozyme. After the enzymatic modification of lysozyme, two structural fractions were distinguished in the composition of the resulting preparations—the monomeric fraction and the peptide fraction. The modified lysozyme exhibited high surface hydrophobicity and high total antibacterial activity despite the decrease in the hydrolytic activity. However, this area requires further specific microbiological investigations to prove the hypotheses about the destructive effect of biopeptides on various microorganisms. Our study showed that the modification of lysozyme with hydrolytic enzymes, especially pepsin, resulted in preparations with very good antioxidative properties. This is a very important and valuable achievement of our research, which will definitely increase the application potential of lysozyme modified in this way.

## Figures and Tables

**Table 1:** The physicochemical characteristics of lysozyme preparations were obtained as a result of its modification in accordance with the procedures of the first variant of the test.

No	Enzyme	pH	Time (min)	Temperature (°C)	Peptide Concentration (%)	Hydrolytic Activity (U/µL)	Hydrolytic Activity (ΔH) (%)	Antioxidant Properties (TE/mgL)
0			60		0 ^a^	21,554 ^k^	0 ^m^	76.7 ^a^
1	Pepsin 0.01%	2		40	67.5 ^c^	9158 ^c^	33.76 ^a^	80.3 ^a^
2				55	75.9 ^d^	5189 ^d^	37.92 ^b^	88.4 ^b^
3				70	88.9 ^e^	3724 ^a^	44.44 ^c^	95.7 ^c^
4	Pepsin 0.02%			40	69.4 ^f^	8052 ^e^	34.62 ^d^	97.3 ^cd^
5				55	80.7 ^g^	5668 ^f^	40.1 ^e^	99.2 ^d^
6				70	91.7 ^h^	3508 ^a^	45.86 ^f^	102.4 ^e^
7	Trypsin 0.02%			40	1.8 ^ab^	18,984 ^b^	0.9 ^g^	96.3 ^c^
8				55	15.1 ^i^	17,757 ^g^	7.6 ^h^	109.4 ^f^
9				70	20.9 ^j^	16,567 ^h^	10.38 ^i^	115.8 ^g^
10	Trypsin 0.03%			40	2.8 ^b^	18,623 ^b^	4.66 ^j^	97.1 ^cd^
11				55	17.5 ^k^	17,332 ^i^	8.6 ^k^	110.5 ^f^
12				70	23.3 ^l^	16,038 ^j^	11.6 ^l^	116.7 ^g^

^a–m^ Different letters in columns denote a significant difference for means at *p* ≤ 0.05. Number of test repetitions: *n* = 5.

**Table 2:** The physicochemical characteristics of lysozyme preparations were obtained as a result of its modification in accordance with the procedures of the second variant of the test.

No	pH	Time (min)	Temperature °C	Peptide Concentration (%)	Hydrolytic Activity (U/µL)	Hydrolytic Activity (ΔH) (%)	Antioxidant Properties (TE/mgL)
0				0 ^b^	21,554 ^b^	0 ^a^	76.7 ^c^
13	2	60	70	67.9 ^c^	9464 ^b^	33.95 ^b^	81.7 ^d^
14		120		61.9 ^d^	8502 ^c^	30.72 ^c^	97.5 ^e^
15		180		55.8 ^e^	8070 ^d^	27.88 ^d^	106.8 ^a^
16	3	60		49.6 ^f^	12,942 ^e^	24.8 ^a^	101.3 ^f^
17		120		45.4 ^g^	12,272 ^f^	22.7 ^e^	107.5 ^a^
18		180		41.1 ^a^	11,922 ^g^	20.5 ^f^	110.9 ^b^
19	4	60		40.6 ^a^	15,606 ^h^	19.8 ^g^	104.8 ^a^
20		120		30.8 ^h^	14,826 ^a^	15.2 ^h^	111.2 ^b^
21		180		21.1 ^i^	14,546 ^a^	10.6 ^i^	113.4 ^b^

^a–i^ Different letters in columns denote a significant difference for means at *p* ≤ 0.05. Number of test repetitions: *n* = 5.

**Table 3:** Microbiological test of lysozyme activity against selected Gram-positive and Gram-negative bacteria.

No (Concentration 3%)	Gram(+) and Gram(−) Bacteria			
	*Listeria innocua*	*Salmonella enteritidis*	*Proteus mirabilis*	*Escherichia coli*
Lysozyme (monomer)	+	−	−	−
13	+	+	+	+
14	+	+	+	+
15	+	+	+	+

− no effect on bacteria, + prolong the lag phase period.

## Data Availability

All data presented in this study are available in the article.

## Sample Availability

Not available.
